# Efficacy and safety of anakinra for undifferentiated autoinflammatory diseases in children: a retrospective case review

**DOI:** 10.1093/rap/rkz004

**Published:** 2019-02-12

**Authors:** Suchika Garg, Karen Wynne, Ebun Omoyinmi, Despina Eleftheriou, Paul Brogan

**Affiliations:** 1 Infection, Inflammation and Rheumatology Section, Great Ormond Street Institute of Child Health, University College London, Great Ormond Street Hospital NHS Foundation Trust, London, UK; 2 Department of Paediatric Rheumatology, Great Ormond Street Hospital NHS Foundation Trust, London, UK; 3 ARUK Centre for Adolescent Rheumatology, University College London, London, UK

**Keywords:** undifferentiated autoinflammatory disease, unclassified autoinflammatory disease, child, anakinra, IL-1 receptor antagonist

## Abstract

**Objective:**

The aim was to carry out a retrospective review of the efficacy and safety of anakinra in paediatric patients with undifferentiated autoinflammatory disease (uAID).

**Methods:**

We carried out a retrospective study of children with uAID at a single quaternary centre. The clinical efficacy of anakinra was evaluated using physician global assessment (PGA) and serological response assessed by levels of serum amyloid A and CRP. Safety was assessed by exploring adverse events, including infection and drug reactions.

**Results:**

This study included 22 patients, 64% females and 36% males of median age 7.1 years (range 0.13–14.11 years), with uAID. The median starting dose of anakinra was 2 mg/kg (range 2–6 mg/kg) and the median duration of treatment 19.6 months (range 0.8–100 months). Before anakinra treatment, the median PGA, on a three-point Likert scale, was 2 (range 1–2), which fell to 1 (range 0–2) within 3 months of treatment. Eight of 22 (36%) patients achieved complete clinical and serological remission; 8/22 (36%) achieved a partial response; and 6/22 (28%) had no response to anakinra. Adverse events included death (3/22, 14%) and allogeneic haematopoietic stem cell transplantation (1/22, 5%). There were no new safety signals, and anakinra was well tolerated overall.

**Conclusion:**

Retrospectively, 72% of children with uAID responded well to anakinra, with 36% achieving full clinical and serological remission within 3 months. This suggests that empirical trials of IL-1 blockade might be warranted in children with uAID. Clear stopping criteria based on predefined parameters should be considered, because non-responders required alternative therapies, facilitated by a definitive molecular diagnosis where possible.

**Key messages**

- Undifferentiated autoinflammatory diseases carry a significant disease burden, and there is a limited therapeutic evidence base.
- Anakinra is efficacious in some undifferentiated autoinflammatory diseases despite the absence of a firm molecular diagnosis.
- Molecular diagnoses for undifferentiated autoinflammatory disease must be continuously re-evaluated as novel pathogenic variants are regularly described.

## Introduction

Autoinflammatory diseases (AIDs) are characterized by inflammation caused by abnormal dysregulation of the innate immune system that leads to periodic fevers, various inflammatory cutaneous manifestations, arthritis, CNS inflammation, inflammatory eye disease, myalgia, serositis, and, in children, delay of growth and puberty [1]. Untreated, all AIDs are associated with risk of organ failure and death from reactive Amyloid A amyloidosis [2]. Clinical trials have demonstrated that IL-1 blockade with the IL-1 receptor antagonist anakinra or the monoclonal antibody against IL-1β canakinumab are highly effective for cryopyrin-associated periodic syndromes (CAPS) and, more recently, have demonstrated efficacy for TNF receptor-associated periodic fever syndrome (TRAPS), mevalonate kinase deficiency (MKD) and colchicine-resistant FMF (crFMF) [3]. Anakinra has recently been licensed in Europe for systemic JIA and adult-onset Still’s disease [4].

These therapeutic advances represent important progress for AID patients, but an important challenge is how to treat patients with unclassified or undifferentiated autoinflammatory disease (uAID), who do not have genetic confirmation of CAPS, TRAPS, MKD or FMF. Published data regarding the use of anakinra for uAID is extremely limited, with one retrospective report pertaining to 11 adults with uAID [5] that suggested anakinra as a viable treatment option. No studies relate to the use of anakinra for uAID in children. The purpose of this study was to describe retrospectively the use of anakinra for paediatric uAID patients.

## Methods

This was a single-centre retrospective review of paediatric patients referred to the autoinflammation service at Great Ormond Street Hospital between January 2009 and January 2018. Inclusion criteria were patients with a diagnosis of uAID, fulfilling a pre-specified definition (see below - Patients section), who received anakinra. Ethical approval was received by the Joint Research and Development department at Great Ormond Street Hospital (reference number: 17IR33). Given that this was a retrospective review of anonymized un-identifiable data, it was exempt from National Health Service (NHS) Research Ethics Committee approval, and consent was not required from individual patients.

### Patients

For the purpose of this study, the diagnosis of uAID required the presence of systemic inflammation, with or without periodic fevers, plus the following key exclusions: a genetic diagnosis of CAPS, FMF, MKD or TRAPS; periodic fever, aphthous stomatitis, pharyngitis and adenopathy syndrome, because this syndrome rarely requires treatment with anakinra [6]; systemic JIA that fulfilled the International League of Association for Rheumatology criteria, because anakinra is now licensed for this indication [4]; and other obvious causes of systemic inflammation, including infection, malignancy or autoimmunity. Clinical features collected were adapted from the autoinflammatory disease activity index (AIDAI) tool [7].

### Outcomes

The physician global assessment (PGA) was used as a primary outcome measure of overall clinical disease activity and was extracted retrospectively from clinical records using a three-point Likert scale: 0 = no to minimal activity; 1 = mild to moderate activity; and 2 = severe activity. The co-primary outcome measure was normalization (or reduction) of CRP (normal range 0–20 mg/l) and serum amyloid A (normal range 0–10 mg/l) 3 months after starting anakinra. These outcomes were divided into three categories as defined below.

#### Complete response (remission)

Efficacy of anakinra was divided into three sub-categories of complete response: clinical remission, PGA = 0/2; serological remission, normal CRP/serum amyloid A levels; and complete remission, clinical and serological remission.

#### Partial response

This was defined clinically as a change from a Likert category to the category below and/or serologically as a ≥50% reduction in CRP, but not in the normal range (0–20 mg/l).

#### No response

This category included patients who failed to meet the criteria for remission or partial response (as above) and patients who died or needed allogeneic haematopoietic stem cell transplantation despite anakinra.

Secondary outcome measures included analysis of adverse effects and laboratory parameters: ESR, haemoglobin concentration, white blood cell count and platelet count; and analysis of the daily prednisolone dose at each of the time points studied.

### Stopping criteria and anakinra treatment duration

Reason(s) for stopping anakinra were collated. These included lack of improvement of PGA and/or acute-phase reactants, or the development of adverse events. Generally, 3 months of anakinra was regarded (in our routine clinical practice) as the minimal duration to gauge therapeutic response; patients with partial response continued treatment for 6 months before terminating it.

### Statistical analyses

Non-parametric descriptive statistics were used for numerical data, and expressed as the median and range. A two-tailed Wilcoxon signed-rank test was used to compare numerical data before and after anakinra to note the presence of a significant difference. Fisher’s exact test was used to compare the response to anakinra with baseline CRP or serum amyloid A levels. A *P*-value < 0.05 was considered statistically significant for all tests. All tests were performed using International Business Machines Statistical Package for Social Sciences version 25.

## Results

### Patient characteristics

Between January 2009 and January 2018, 54 children treated for rheumatological conditions at Great Ormond Street Hospital received anakinra for miscellaneous diagnoses. Of these, 32 were excluded from our study because they did not meet the inclusion criteria for uAID as defined above. There were 64% females (14/22) and 36% males (8/22). The median age at symptom onset was 0.61 years (range 0–13.5 years), with 14/22 children presenting in the first year of life. Consanguinity was present in 4/22. A family history of inflammatory rheumatological conditions was present in 7/22.

### Baseline clinical features

Clinical features are summarized in Supplementary Table S1, available at *Rheumatology Advances in Practice* online. CSs were used in 11/22 patients at anakinra introduction. NSAIDs were used in 3/22. DMARDs were prescribed to 13/22 children: MTX (*n* = 5), CSA (*n* = 3), AZA (*n* = 4), and MMF (*n* = 1). Anakinra was commenced because of lack of adequate clinical response to these previous treatments. The median age at commencement of anakinra was 7.1 years (range 0.13–14.11 years). Immediately before starting anakinra, the median PGA was 2 (range 1–2), CRP was raised in 13/22 patients [median 39 mg/l (range 5–344 mg/l)], and 4/22 patients did not have serum amyloid A recorded, while 10/18 patients had elevated serum serum amyloid A levels [median 122 mg/l (range 2–637 mg/l)].

### Response to anakinra

Twenty-one of 22 children started anakinra 2 mg/kg/day injections. Patient 4 started 6 mg/kg injections, owing to an episode of secondary haemophagocytic lymphohistocytosis at the time of commencement (Table 1). This was tapered to 1 mg/kg/day as the disease went into remission. Twelve of 22 patients required increased anakinra doses to control their disease, with a maximal increase of 6 mg/kg for patient 3. All children received anakinra daily, with the exception of two patients who were on alternate-day injections: patient 11 was on peritoneal dialysis, hence the reduced dosage; and patient 18 was in remission, which led to a switch from daily to alternate-day injections.

**Table 1:** Genetic findings, baseline inflammatory markers and treatment responses

Patient identity	Gene identity	Amino acid change	Zygosity	Variant pathogenicity class [8]	Genetic test	Final diagnosis	Baseline CRP	Baseline serum amyloid A	Response to anakinra	Final treatment—outcome at last follow-up
1	*LPIN2*	P626S/S203F [8]	Comp. Het	4	VIP	Possible Majeed syndrome	155	279	CR	Anakinra—remission
2	—	—	—	—	WGS (pending)	uAID	41	168	PR	Anakinra—active disease
3	—	—	—	—	VIP	uAID	183	—	CR	Anakinra—remission
4	—	—	—	—	VIP	uAID	250	—	CR	Remission
5	—	—	—	—	VIP	uAID	130	237	NR	Deceased; MAS
6	*PSMB4*	P16Sfs*45 [9]	Het	5	WES	CANDLE syndrome	5	2.9	NR	Deceased; multi-organ failure from CANDLE syndrome, not on anakinra time of death
	*PSMB9*	GI65D [9]	Het							
7	*WDR1*	L293F [10]	Homo	5	WES	PFIT syndrome	5	6	NR	Deceased; multi-organ failure from PFIT syndrome, not on anakinra time of death
8	*NOD2*	D512Y [8]	Het	5	VIP/WES	Blau’s syndrome	344	55	PR	Canakinumab, colchicine— remission
9	—	—	—	—	WES	uAID	50	237	NR	Allogeneic haematopoietic stem cell transplant—cure
10	*PSMB4*	P16Sfs*45 [9]	Het	5	WES	CANDLE syndrome	165	—	PR	Baricitinib—remission
	*PSMB9*	GI65D [9]	Het							
11	—	—	—	—	VIP	uAID	10	5	CR	Anakinra—remission
12	—	—	—	—	WES	uAID	37	146	CR	Anakinra—remission
13	—	—	—	—	Sanger	uAID	42	374	NR	Infliximab, AZA—remission
14	—	—	—	—	VIP	uAID	5	10	PR	Tocilizumab—remission
15	—	—	—	—	VIP	uAID	21	76	NR	Colchicine, tocilizumab— remission
16	*PRG4*	Q1305X	Homo	5	Sanger	CACP syndrome	5	4	PR	MTX—remission
17	*ADAM17* (+; VUS)	I50V/R215I	Het	4	WES	uAID	15	—	PR	Remission
18	—	—	—	—	Sanger	uAID	54	374	CR	Anakinra—remission
19	*PLCG2*	Y482H/N571S [8]	Het	4	VIP	uAID	5	3.4	PR	Remission
20	—	—	—	—	VIP/WES	uAID	15	345	CR	Canakinumab^a^—remission
21	—	—	—	—	WES	uAID	49	637	PR	Canakinumab—remission
22	—	—	—	—	WES	uAID	19	98	CR	Anakinra—remission

Genetic testing was done to provide a unified diagnosis of these uAID patients, with the specific gene identity and the amino acid change found for eight patients (patients 1, 6, 7, 8, 10, 16, 17 and 19). Pathogenicity of the variants was as described in [8]. Genes were screened using Sanger and/or next generation sequencing (NGS); targeted NGS vasculitis and inflammation gene panel (VIP [8]), whole exome sequencing (WES) and whole genome sequencing (WGS). NGS was available via P.B. (WES or VIP) or via the national 100 000-genomes project (WGS). The final disease label is the disease label at last clinical follow-up. The therapies patients were undergoing at the last clinical follow-up highlight the wide range of therapies patients were on after failing anakinra. Anakinra—remission represents patients who are currently on anakinra with their disease in remission. Remission represents those who stopped anakinra because their disease was controlled (and in remission) without it. ^a^In patient 20, anakinra was efficacious but the patient was switched to canakinumab owing to inability to cope with daily injections. CACP syndrome: camptodactyly, arthropathy, coxa vara, pericarditis; CANDLE syndrome: chronic atypical neutrophilic dermatosis with lipodystrophy and elevated temperature syndrome; CAPS: cryopyrin-associated periodic syndrome; CR: complete response; CRMO: chronic recurrent multifocal osteomyelitis; MAS: macrophage activation syndrome; NR: no response PFIT: periodic fever, immunodeficiency and thrombocytopenia; PR: partial response; uAID: unclassified or undifferentiated autoinflammatory disease; zygosity: Comp. Het: compound heterozygous; Het: heterozygous; Homo: homozygous.

### *Physician global assessment*

PGA was scored on a three-point Likert scale at different intervals after starting anakinra. At the time of starting anakinra, 77% (17/22) patients had PGA = 2 (severe disease activity), and 23% (5/22) had PGA = 1 (mild to moderate activity). The number of patients with PGA = 2 decreased over the course of anakinra treatment. Within 3 months, 45% (9/20) of patients had PGA = 0 (minimal disease activity), and this increased to 55% (10/18) within 6 months (Supplementary Table S2). The increased number of patients with PGA = 2 (27%, 6/22 patients) at the last follow-up was because this time point included patients who discontinued anakinra owing to intolerance or ineffectiveness and included 3/22 patients who died (14%; patients 5, 6 and 7), 1/22 patients who received allogeneic haematopoietic stem cell transplantation (5%; patient 9) and another patient with severe tonsillitis and periorbital cellulitis, which necessitated stopping anakinra (patient 17; Table 1).

**Table 2:** Results of physician global assessment and serological markers at various time points throughout treatment

	Baseline (*n* = 22)	3 months (*n* = 20)	6 months (*n* = 18)	Last visit on anakinra [median 19 months (range 1–100 months)] (*n* = 22)	Last visit off anakinra [median 35 months (range 11–153 months)] (*n* = 15)
Primary outcome measures					
Physician global assessment	2 (1–2)	1 (0–2)	0 (0–2)	0 (0–2)	0 (0–2)
CRP (mg/l)	41 (5–344)	5 (1–26)	5 (1–53)	6 (1–350)	5 (1–20)
		***P*** = **0.001**	***P*** = **0.007**	*P* = 0.08	***P*** = **0.018**
Serum amyloid A (mg/l)	146 (3–637)	4 (3–216)	3 (1–32)	5 (3–58)	3.3 (3–4)
		***P*** = **0.012**	*P* = 0.075	***P*** = **0.028**	*P* = 0.109
Secondary laboratory outcome measures					
ESR (mm/h)	50 (3–100)	10 (1–52)	17 (4––50)	14 (2–110)	7 (2–110)
		***P*** = **0.023**	***P*** = **0.025**	*P* = 0.086	***P*** = **0.017**
Hb (g/l)	110	120	121	122	130
	(76–126)	(90–145)	(107–129)	(70–155)	(96–135)
		***P*** = **0.002**	***P*** = **0.021**	***P*** = **0.02**	***P*** = **0.021**
WBC count (×10^9^/l)	11 (3–7)	8 (3–14)	7 (3–13)	9 (2–24)	6 (3–24)
		*P* = 0.088	*P* = 0.173	*P* = 0.233	*P* = 0.401
Platelets	393 (107–615)	321	385	298	312
		(196–653)	(130–530)	(204–493)	(202–430)
		*P* = 1	*P* = 0.176	*P* = 0.363	*P* = 0.674
	**Baseline (*n*** = **11)**	**3 months (*n*** = **11)**	**6 months (*n*** = **8)**	**Last visit on anakinra [median 19 months (range 1–100)] (*n*** = **8)**	**Last visit off anakinra [median 35 months (range 11–153)]**
Daily prednisolone dose analysis					
Prednisolone (mg/kg)	0.33	0.32	0.30	0.08	N/A
	(0–1.43)	(0.06–2)	(0–0.71)	(0–1.25)	
		*P* = 0.241	*P* = 0.161	*P* = 0.398	

All results are displayed as the median (range). The *P*-values represent comparison at 3 and 6 months and last clinical follow-up (on and off anakinra) compared with the baseline for each variable using the two-tailed Wilcoxon signed-rank pair test; *P*-values < 0.05 were considered significant and are highlighted in bold. The daily prednisolone dose (in milligrams per kilogram body weight) was analysed at baseline, 3 and 6 months after commencing anakinra and the last visit on anakinra. Physician global assessment ranges from zero to two, where two represents maximal disease activity and zero represents minimal disease activity. ESR, normal range 0–10 mm/h; N/A: not applicable (the daily prednisolone dose was not analysed after patients stopped anakinra, because the primary aim of analysing the daily doses was to looking for a steroid*-*sparing effect of anakinra); serum amyloid A, normal range 0–10 mg/l; WBC count: white blood cell count, normal range 4 × 10^9^ to 11 × 10^9^/l.

### Acute phase reactants and serological markers

Ninety per cent (18/20) of patients achieved a normal CRP within 3 months, and 94% (17/18) had achieved this within 6 months of treatment. Seventy per cent (14/20) of patients achieved a normal serum amyloid A level within 3 months, while 10% (2/20) had a ≥50% decrease in serum amyloid A levels from baseline, which had not normalized. Within 6 months, 72% (13/18) had achieved normal serum amyloid A levels.

Two patients, patients 9 and 13, ended treatment before the 3 months mark owing to an incomplete response to anakinra. Two more patients (patient 14, with inadequate symptom control; and patient 20, for whom anakinra worked but who was switched to canakinumab, patient choice) ended treatment before the 6 months follow-up. Given that these patients stopped anakinra before the 3 and 6 months follow-ups, they were excluded from the respective time point analyses, and it was assumed that patients 9, 13 and 14 had failed treatment.

Efficacy results at 3 and 6 months after anakinra commencement are summarized in Tables 1 and 2. PGA and CRP improved at 3 and 6 months, and PGA at the last visit was also significantly improved while still on anakinra. A similar trend was observed for serum amyloid A, ESR and haemoglobin. There were non-statistically significant changes in white blood cell and platelet counts. Baseline CRP or serum amyloid* *A did not predict response to treatment with anakinra (Table 1; Fisher’s exact test, *P*-value = non-significant, data not shown).

### Safety

Three main subtypes of adverse effects recorded in this study were as follows: injection-site reactions (erythema and pain around injection site [11]), infections and neutropenia. Fifteen of 22 had a median of one adverse event (range 0–2): infection (*n* = 8); neutropenia (*n* = 7); and injection-site reaction (*n* = 5). Twelve events in 10/22 patients were deemed serious and required hospital admission: three patients had painful injection-site reactions requiring presentation to their local hospital, although none of these ultimately required specific intervention or cessation of treatment. Serious infections requiring hospital intervention were as follows: presumed viral infection (no further details provided) with disease flare (*n* = 1); urinary tract infection and concomitant varicella zoster virus infection (*n* = 1); presumed viral upper respiratory tract infection (*n* = 1); suspected sepsis (no organism isolated) and disease flare (*n* = 1); and orbital cellulitis (no organism isolated; *n* = 1; Supplementary Table S3, available at *Rheumatology Advances in Practice* online). Neutropenia requiring presentation to the patients’ local hospital was detected on blood monitoring in three patients, all self-limiting (but no further details were available). Eight events in 6/22 patients were deemed not serious and did not require hospitalization (Supplementary Table S3, available at *Rheumatology Advances in Practice* online).

Three patients died. One died while on anakinra (patient 5; Table 1) from macrophage activation syndrome in the context of the unclassified autoinflammatory disorder, and two patients died from multiorgan failure attributable to their underlying disease having stopped anakinra previously (patients 6 and 7; Table 1).

### Duration of treatment and discontinuation

At the last clinical follow-up, 7/22 patients were still on anakinra treatment, with 6/7 in remission. The median treatment duration for the other 15 patients was 5.1 months (range 0–100 months). The reasons for discontinuation included the following: lack of efficacy (8/15, 53%); death (3/15, 20%); disease in remission (2/15, 13%); intolerance (1/15, 7%); and change in diagnosis (1/15, 7%) (Supplementary Figure S1, available at *Rheumatology Advances in Practice* online).

### Diagnostic impact of further genetic testing

At the start of the study in 2009, standard fever gene screening for patients with periodic fevers focused only on TRAPS, CAPS, MKD and FMF, thus patients with negative screening for these diseases were designated as uAID. Over the course of the study, however, the patient cohort underwent additional genetic testing (Sanger and/or next generation sequencing), with diagnostic impact on 8/22 (38%) patients. The final diagnoses are summarized in Table 2.

## Discussion

The aim of this study was to explore retrospectively the safety and efficacy of anakinra in uAID paediatric patients, because these data are currently lacking. Retrospectively, we observed that 36% (8/22) of patients achieved complete remission within 3 months of starting anakinra and remained in remission at their last clinical follow-up, indicating that the initial response to anakinra was a reliable predictor of longer-term efficacy. Thirty-six per cent (8/22) had a partial response. The remaining 28% (6/22) patients had no discernible response to anakinra. Baseline CRP or serum amyloid A did not predict response to anakinra. Empirical trials of biologics or other immunomodulators are thus justifiable despite a lack of high-level evidence, because our observation of significant mortality (14%) or the need for haematopoietic stem cell transplantation (5%) demonstrated the severity of uAID in some patients.

Anakinra might not be effective for all uAID patients; in this series, 15/22 stopped anakinra for the following reasons: lack of efficacy (53%); death (20%); remission (13%); intolerance to daily injections (7%); and change of diagnosis (7%). Further genetic testing of this series of uAID patients resulted in a definitive molecular diagnosis in 36% of the patients (patients 1, 6, 7, 8, 10, 16, 17 and 18), comparable to a previous report on the clinical impact of next generation sequencing in such patients [8], with therapeutic implications in some patients. With regard to safety, unlike previous studies, the most common side effect was not injection-site reactions [11], but infection. Previous studies have reported that IL-1 blockade leads to increased susceptibility to infections [12]. The frequency of injection-site reaction was perhaps lower in this cohort owing to concomitant oral and/or topical CSs, which reduce the severity and incidence of injection-site reactions [13]. Overall, anakinra was well tolerated, with no new safety signals.

Our study is limited by all the caveats around a retrospective case series and is thus certainly subject to bias. Simple parameters, such as the PGA, and serological responses (CRP and serum amyloid A ) have been used in other clinical trials of autoinflammation [14, 15] and have thus faced validation in this context. Unfortunately, patient-reported quality-of-life data were not collected in this retrospective study, which is a limitation of our study.

Advances in next generation sequencing technologies have had significant clinical diagnostic impact for patients with autoinflammation [8] and in the future will be key to realizing the vision of precision medicine of more targeted treatments for uAID patients. In the meantime, empirical trial of IL-1 blockade with anakinra is arguably justifiable for paediatric patients with uAID, because a significant proportion will respond, and this is a safe approach.

## Supplementary Material

Supplementary Data
