# Supplementary figures and images for: Efficacy and safety of anakinra for undifferentiated autoinflammatory diseases in children: a retrospective case review
Source: Rheumatol Adv Pract. 2019 Feb 12;3(1):rkz004. doi: 10.1093/rap/rkz004 (PMC6649913; doi:10.1093/rap/rkz004)

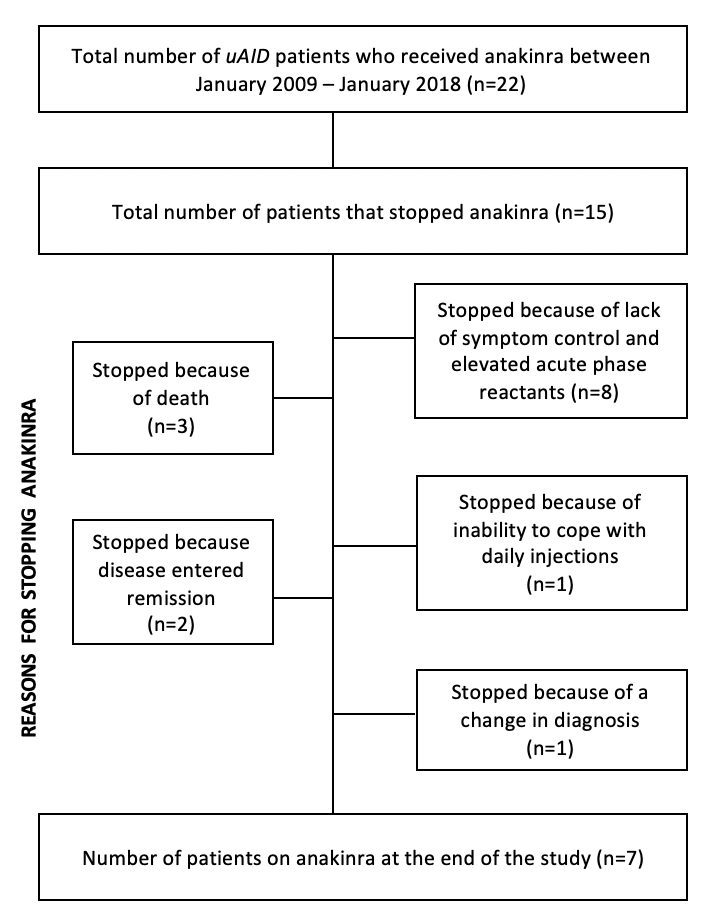

Supplement: Supplementary Data [file rkz004_supp.png]
